# Qualitative Insights From Practicing Speech and Language Therapists on Key Textural Attributes of Transitional Foods for Dysphagia Management

**DOI:** 10.1111/jtxs.70064

**Published:** 2026-01-30

**Authors:** Seh Ling Kwong, Julia Mei Wan Lee, Suk Meng Goh, Valerie Puay Cheng Lim, Simeon Dobrev Stoyanov, Katsuyoshi Nishinari

**Affiliations:** ^1^ Singapore Institute of Technology Singapore Singapore; ^2^ Tan Tock Seng Hospital Singapore Singapore; ^3^ Glyn O. Phillips Hydrocolloid Research Centre, School of Bioengineering and Food Science, Hubei University of Technology Wuhan China

**Keywords:** dysphagia, focus group, IDDSI, speech and language therapy, texture, transitional foods

## Abstract

Transitional foods, as defined by the International Dysphagia Diet Standardization Initiative (IDDSI), are foods that change texture with moisture or temperature. They show promise for dysphagia management, but their clinical use is limited due to unclear guidelines and insufficient understanding of their transitional characteristics. This study aimed to identify key textural attributes of transitional foods through qualitative insights from 15 practicing speech and language therapists in Singapore. Four focus group discussions were conducted, during which participants also evaluated 10 food samples using sensory testing and supplemented by parallel IDDSI testing. Thematic analysis revealed key criteria: rapid transition time (ideally 5–10 s), even texture change, minimal force for breakdown, cohesive bolus formation, and absence of stickiness or mixed consistencies. Foods transitioning with minimal effort, such as baby puff, baby milk biscuit ball, and baked meringue samples, were considered transitional (> 90% participant agreement), while those requiring greater mechanical manipulation or showing uneven transitions, such as cheese puff and chiffon cake, were deemed less suitable (≤ 50% agreement). Discrepancies between sensory evaluations and IDDSI test outcomes, such as for tofu pudding and gelatin jelly, highlighted limitations in current testing protocols, which use a 1‐min observation period at ambient temperature and do not account for total mechanical effort. Participants also emphasized the importance of age‐appropriate appearance, localized flavors, and practical considerations for implementation. These findings support refining definitions and testing methods for transitional foods and provide foundational data to guide future product development and standardization in dysphagia care.

## Graphical abstract

The desirable attributes of transitional foods include quick melting, minimal force to breakdown, even texture transitions, low stickiness, and a low degree of mixed consistencies. Instrumental testing methods for transitional foods require further development.

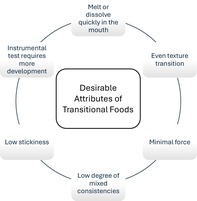

## 1 Introduction

Dysphagia refers to an observed or perceived difficulty in moving food from the mouth to the esophagus (Cook and Kahrilas 1999). It has been recognized as a geriatric syndrome with a high prevalence among older adults (Baijens et al. 2016). The prevalence of dysphagia ranged from 27% of older persons in the community (Serra‐Prat et al. 2011) to 91% in older hospitalized patients with community‐acquired pneumonia (Almirall et al. 2013). In this population, dysphagia can lead to severe complications that significantly impact health, nutritional status, functional ability, morbidity, mortality, and overall quality of life (Baijens et al. 2016).

Various studies have shown that swallowing is a complex process encompassing neurological, cognitive, motor, and sensory aspects (Hamdy et al. 1999; Lowell et al. 2008; Winchester and Winchester 2015). Notably, dysphagia has been associated with cognitive impairment (Dehaghani et al. 2021; Jo et al. 2017; Maniaci et al. 2024; Moon et al. 2012). Swallowing difficulties can ultimately lead to a vicious cycle: reduced food intake results in reduced nutrient intake, which in turn impairs immunity, contributes to cognitive impairment, and subsequently further affects food intake (Calder et al. 2018). With aging, susceptibility to dysphagia is further heightened by factors such as sarcopenia (a loss of muscle mass and function), leading to sarcopenic dysphagia (Fujishima et al. 2019), and oral hypofunction, which reflects declines in oral motor and masticatory function (Minakuchi et al. 2018).

Texture‐modified diets are a common clinical approach for managing dysphagia (Hansen et al. 2022; Rothenberg and Wendin 2015). In these diets, the texture, consistency, and viscosity of food and drinks are adjusted to reduce the risk of aspiration and choking (Wu et al. 2020). Some studies have shown that institutionalized elderly individuals with dysphagia increased their oral intake and body weight when provided with texture‐modified foods (Germain et al. 2006; Reyes‐Torres et al. 2019).

**FIGURE 1:**
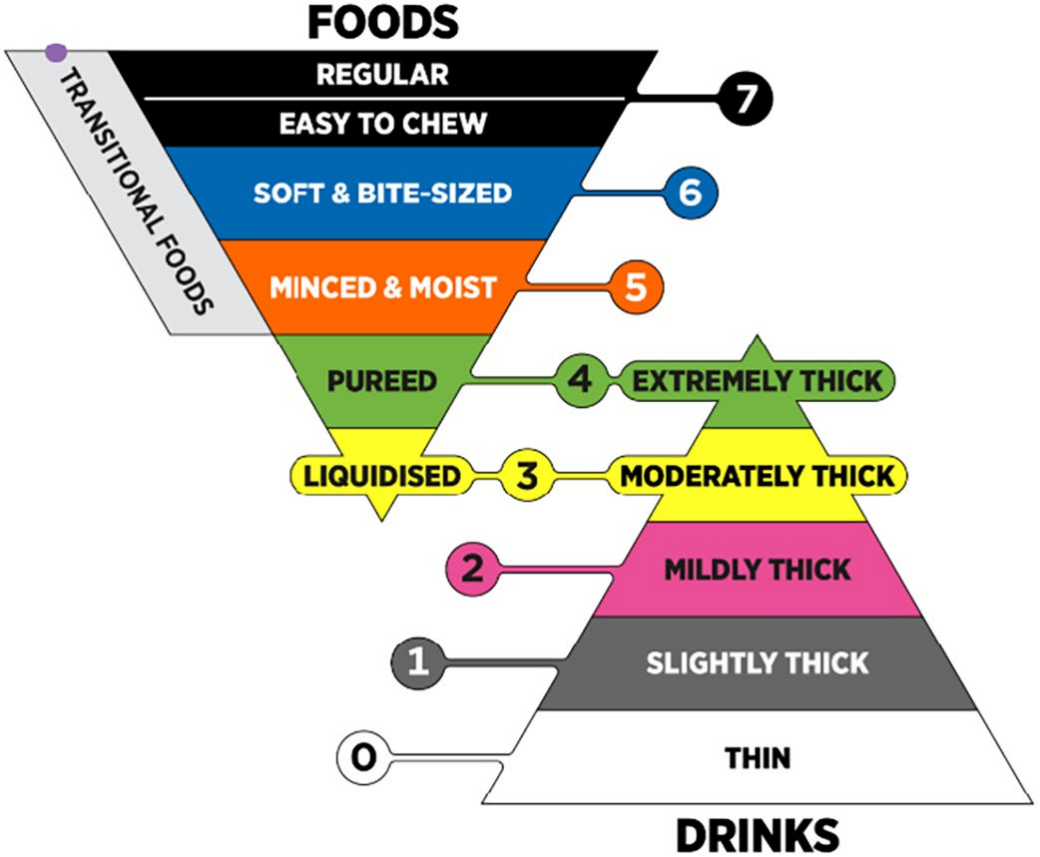
The IDDSI Framework. The International Dysphagia Diet Standardization Initiative 2019@https://iddsi.org/framework. Licensed under the CreativeCommons Attribution Sharealike 4.0 License https://creativecommons.org/licenses/by‐sa/4.0/legalcode. Derivative works extending beyond language translation are not permitted.

To standardize the terminology and definitions relating to texture‐modified foods and drinks, the International Dysphagia Diet Standardization Initiative (IDDSI) framework (Figure 1) has been developed and implemented in many countries (Cichero et al. 2017; International Dysphagia Diet Standardisation Initiative 2019a). The IDDSI framework consists of eight levels covering various consistencies of liquids (Levels 0–4) and foods (Levels 3–7). Specifically, Level 3 is used both for Liquidized foods and Moderately Thick fluids, while Level 4 is used both for Pureed food and Extremely Thick fluids. Pureed foods refer to (otherwise solid) foods, which are blended into a homogeneous texture. Consuming pureed foods is a challenge as they are typically unappetising and unrecognizable (Stahlman et al. 2001). It is therefore not surprising that much research attention has been devoted to making pureed foods more attractive for consumption, including utilizing 3D food printing technology (Guo et al. 2023; Lorenz et al. 2022; Molimi et al. 2025; Pant et al. 2021; Rathi et al. 2025).

Under the IDDSI framework, there is a distinct categorization of transitional foods represented by a sidebar spanning Level 5 (Minced and Moist) to Level 7 (Regular). Transitional foods are defined as foods that “start as one texture but change into another texture when moisture like water or saliva is added or when a change in temperature occurs” (International Dysphagia Diet Standardisation Initiative 2019b). The inclusion of a transitional foods category was endorsed by 40% of 3190 respondents across 57 countries in the draft IDDSI framework stakeholder survey, with clinicians managing pediatric and developmentally disabled populations highlighting the need for a category encompassing “meltable” or “dissolvable” solid foods (Cichero et al. 2017). This feedback led to the current sidebar classification spanning Levels 5–7. Examples of such foods include wafers, shortbread, potato crisps, Cheeto Puffs, Rice Puffs, and ice cream (International Dysphagia Diet Standardisation Initiative 2019b). Transitional foods are also recognized within Japan's Universal Design Foods (UDFs) classification (Japan Care Food Conference, n.d.), which follows the IDDSI definition. The UDF system was established to guide the labeling of food products developed for older adults by participating companies, including food toppings and standard food items.

In early childhood, transitional foods are used to bridge the gap between liquids and solids (Cichero 2017; Dovey et al. 2013; Gisel 1991; Harris and Coulthard 2016). Although guidelines emphasize the importance of advancing textures in a timely manner (Schwartz et al. 2011), no standardized marketing or labeling exists for products with varying degrees of texture. Awadalla et al. (2018) found that many “first finger foods” contravened the recommendations by the American Academy of Pediatrics. Separately, Tan et al. (2022) reported wide textural variations among infant biscuits/wafers and extruded rice snacks. The authors concluded that there are opportunities to design food textures that encourage chewing and align solid breakdown with oral motor development.

It is widely acknowledged that food consumption is a rich, multisensory, and dynamic embodied experience, deeply intertwined with cultural meaning and emotional well‐being (Dantec et al. 2021; Lee 2023; Nishinari et al. 2024; Schifferstein 2021). However, for individuals with dysphagia, this complex experience is fundamentally constrained by the physiological imperative of safety. Transitional foods, which retain the appearance of regular foods, offer the potential to access the cultural and emotional benefits of eating in a safe and manageable manner. Yet, before the sensory, quality‐of‐life, and neurological dimensions can be optimized, there is a critical need to establish a rigorous framework of measurable safety attributes. Defining these objective clinical parameters is not a reduction of the eating experience but the essential ethical prerequisite for ensuring safe access to it.

Research on transitional foods for individuals with dysphagia is sparse. Recently, Barewal and colleagues (Barewal et al. 2021; Bayne et al. 2022; Bruno et al. 2025) examined the behavioral properties of commercially available snack products that are known as transitional foods. Their results highlight the potential to enhance patient safety, quality of life, and nutritional intake. However, gaps persist in understanding the breakdown and lubrication of these foods during oral processing, specifically concerning dissolution rates, bolus formation, and performance during the actual swallow. Furthermore, there is a lack of clear clinical guidelines and standardization for evaluating the properties of transitional foods.

To address these gaps and achieve a clearer definition of the characteristics that make transitional foods suitable for speech therapy practice, a series of focus group discussions was conducted with registered speech therapists in Singapore. In these sessions, participants also tasted and evaluated food samples to describe their characteristics in the context of transitional foods. This manuscript presents the methodology and the insights gained from these discussions.

## 2 Materials and Methods

### 2.1 Recruitment of Participants

Ethical approval for this study was granted by the Singapore Institute of Technology Ethics Committee, reference number RECAS‐0318. All participants of the focus group discussions consisted of Singapore Allied Health Professions Council (AHPC)‐registered speech and language therapists (SLTs) who had experience with managing clients with swallowing or feeding difficulties within the last 5 years. Participants who expressed their interest in participating in the focus group discussion through an online survey^1^ were contacted directly by the study team. Additional participants were recruited through contacts of existing participants. Each participant received 120 Singapore Dollars as remuneration for their time and effort.

A total of 15 SLTs participated, and four discussion groups consisting of two to five participants each were organized. The focus groups were grouped by institutional setting and geographical location. One of the focus groups consisted of participants from the same hospital, while the other three were from mixed institutions.

### 2.2 Samples

As part of the discussion, participants performed food tasting. Ten samples were provided, including eight commercially available products and two baked meringue samples prepared by the project team. The specific products, along with their approximate sizes, are listed in Table 1. Samples were presented as typically consumed. The baby milk biscuit balls were largely spherical with a flattened bottom, while the baked meringue samples were piped into spherical drops with flattened bottoms. The communion wafer and baby puffs were presented as‐is, whereas the cheese puffs, chiffon cake, and milk chocolate were cut into cubes or cuboids. The gelatin jelly and tofu pudding were served in small cups, and participants were instructed to consume half a teaspoon (kept within a 1.5 cm × 1.5 cm size where possible to match IDDSI transitional food testing practices; International Dysphagia Diet Standardisation Initiative 2019b). Notably, the tofu pudding consisted of two components—the pudding and a syrup component.

**TABLE 1:** Food samples used during the focus group discussion sessions.

Food sample	Brand	Approximate sample size
Baby milk biscuit ball	Wang Wang Xiao Man Tou	1.5 cm diameter × 1 cm height
Baked meringue #1	Produced in the laboratory	1.5 cm diameter × 1 cm height
Baked meringue #2	Produced in the laboratory	1.5 cm diameter × 1 cm height
Communion wafer	E‐commerce retailer	3.0 cm diameter × 0.2 cm height
Baby puff	Gerber	1.5 cm length × 0.5 cm height
Cheese puff	Cheezels	1.5 cm × 1.5 cm × 0.5 cm height
Chiffon cake	Local bakery (Harriann's Nonya Table)	1.5 cm × 1.5 cm × 1.5 cm
Milk chocolate	Cadbury's	1.5 cm × 1.5 cm × 0.9 cm height
Gelatin jelly	Tortally	Half a teaspoon
Tofu pudding	Fortune	Half a teaspoon

### 2.3 Procedure for Discussion

Each focus group met in a private room. After introductions, participants provided written consent. Discussions lasted 1.5–2 h and were guided by a researcher using a question guide, encouraging interaction and follow‐up questions. Sessions were audio recorded, and field notes were taken by one team member. Due to a technical issue, the second focus group's recording was lost; field notes were used to identify themes.

The discussion comprised four parts (see Appendix in the Supporting Information for details):

- Part 1: Introduction and understanding of transitional foods (four questions).
- Part 2: Sample tasting.
- Part 3: Characteristics of transitional foods (5 questions).
- Part 4: Recommendations (1 question).

In Part 1, participants were provided with a list of food examples compiled from an earlier online survey^1^ of 58 SLTs in Singapore. They discussed whether they considered each example to be a transitional food.

### 2.4 Sample Tasting

In Part 2, participants evaluated 10 food samples in the fixed order listed in Table 1. Each participant was provided a single data collection form, featuring one row per sample, to record their evaluation:

For each sample, the following procedure was performed:

1. At the facilitator's prompt, participants placed the sample in their mouth. They were instructed not to masticate but could gently press the sample against the hard palate with their tongue. The facilitator started a timer that was visible to all participants.
2. Participants processed the sample at their own pace and recorded the time point at which the sample had “dissolved/melted” in their mouth and was a ready‐to‐swallow bolus.
3. Participants were also instructed to note the transition behavior of the sample and further recorded their evaluation of:
  - whether the food was transitional (yes/no),
  - the initial IDDSI level (right as the sample was placed in the mouth),
  - the final IDDSI level (at the point when the sample was a ready‐to‐swallow bolus)
4. A facilitated discussion was then conducted, guided by these questions:
  - Would you consider this food transitional? Why or why not?
  - Please describe how the food transitioned in the mouth

Due to the participants' clinical experience and daily application of IDDSI standards in professional practice, formal training on IDDSI level identification was not conducted. During tasting, participants estimated the initial and final IDDSI levels by clinical observation where appropriate (visual or tactile as the sample was picked up or spooned) and in‐mouth sensory judgment. This approach mirrored the current bedside practice for SLTs and constituted a subjective, expert clinical estimation.

### 2.5 IDDSI Fork Pressure Test for Transitional Foods

To avoid biasing discussion of design‐relevant attributes, the formal IDDSI test for transitional foods (International Dysphagia Diet Standardisation Initiative 2019b) was conducted separately by the research team and reported independently. Samples were cut to nominal dimensions of 1.5 × 1.5 cm, where possible, as per IDDSI test directions. One millimeter of water was added to each sample and left for 1 min. Subsequently, one researcher performed the standard fork pressure test—where a fork was used to press down on the sample with a force until the nail blanched white. An AHPC‐registered SLT on the research team also reviewed the results through photographs.

## 3 Results

### 3.1 Participants

Among the 15 participants, 11 were from an acute hospital setting, three were from a community hospital setting and one was from a private clinic. Twelve participants had experience managing adult clients only, one participant had pediatric experience only, and two participants had experience managing both adult and pediatric clients. Participants reported that, in routine practice, they do not apply IDDSI transitional food tests at bedside when trialing foods; rather, they judge suitability through clinical observation and in‐mouth sensory estimation.

### 3.2 Sample Tasting

**FIGURE 2:**
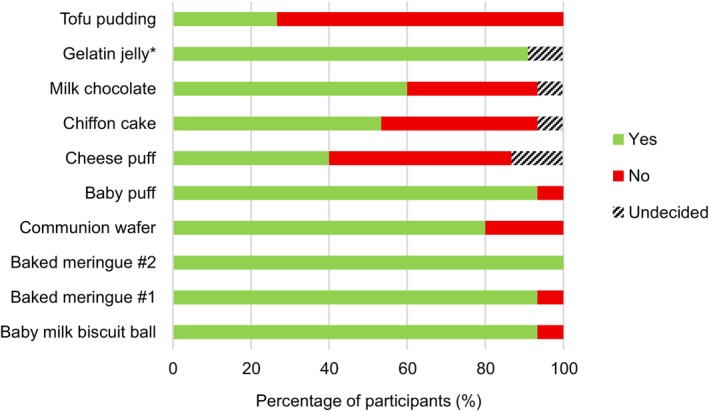
Classification of food samples as transitional or not. *Not all participants tasted the gelatin jelly due to dietary restrictions and spoilage during transportation (*n* = 11).

Figure 2 summarizes participants' evaluations of the food samples, categorizing them as transitional or nontransitional foods following tasting (without mastication). The majority classified baby milk biscuit balls, both baked meringue samples, baby puffs, and gelatin jelly as transitional. Cheese puffs, chiffon cake, and milk chocolate elicited less consensus among panelists, receiving ratings across all three categories of “yes,” “no,” and “undecided.” Tofu pudding was the least frequently classified as transitional.

**FIGURE 3:**
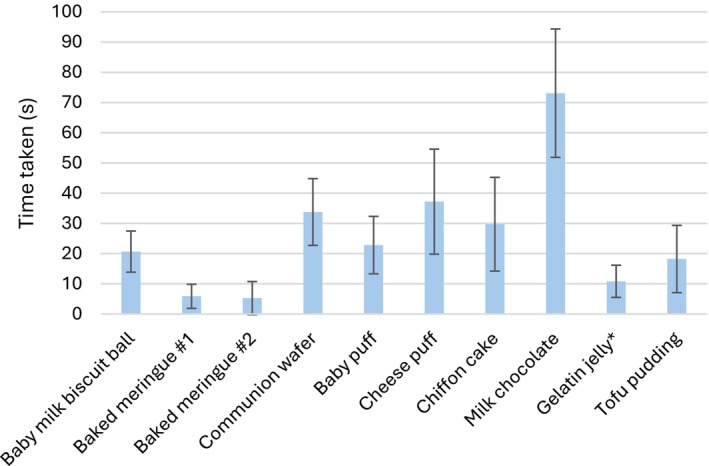
Average time taken to swallow the food samples.

Among samples judged transitional, the two baked meringues and the gelatin jelly took the shortest time (< 20 s) to swallow (Figure 3). The baby milk biscuit balls and baby puffs required slightly longer times compared to tofu pudding, which was not classified as transitional. The time to swallow the communion wafer was like those of cheese puffs and chiffon cake. Despite being rated transitional by more than half of the participants, milk chocolate took the longest to swallow (> 1 min).

**FIGURE 4:**
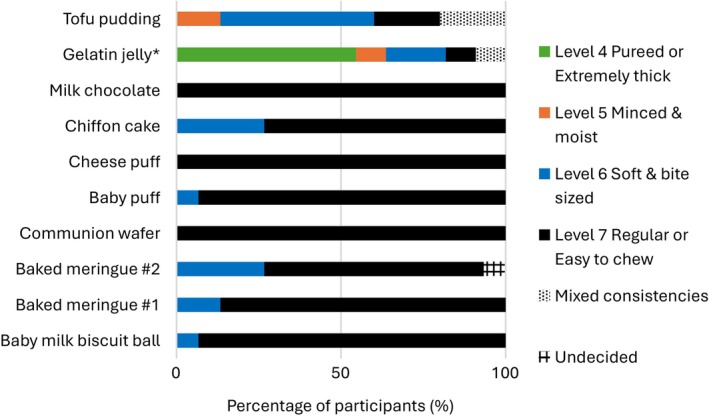
Participants' perception of the initial texture of the food samples based on IDDSI categorization.

**FIGURE 5:**
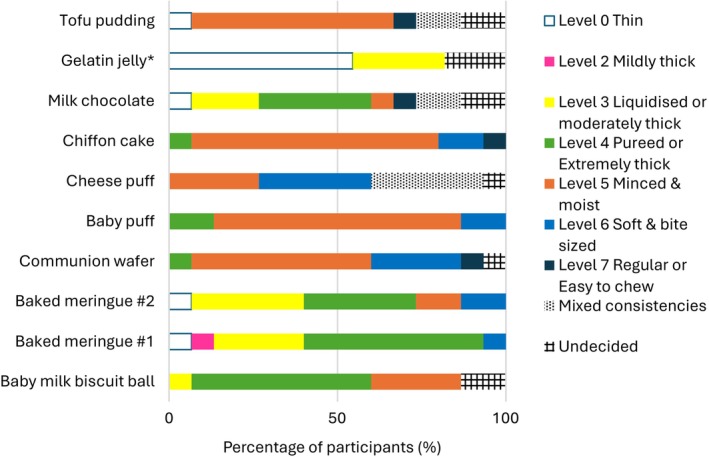
Participants' perception of the final texture of the food samples based on IDDSI categorization.

Figures 4 and 5 show participants' evaluations of the initial and final texture (just prior swallowing) of the samples. Except for the two gel‐like samples of gelatin jelly and tofu pudding, the initial textures of the other foods were classified by many participants as Level 7 Regular or Easy to Chew. One participant was undecided over the initial texture of baked meringue #2 and described it as in between Level 6 Soft and Bite‐sized and Level 7 Easy to Chew. There were clear difficulties in assigning an initial texture to the gel‐like samples, as the ratings ranged from as high as Level 7 Easy to Chew to Level 4 Pureed diet or Extremely Thick fluids, including even one “mixed consistencies” classification (i.e., mix of solid and fluid consistencies in one bolus or mouthful).

For all samples, there was a shift to lower IDDSI levels in the final texture compared to the initial texture. The final texture of milk biscuit balls, both meringues, milk chocolate, and gelatin jelly was generally rated as Level 4 Pureed/Extremely Thick or below, indicating a transition from a more solid‐like to a more liquid‐like consistency. In contrast, the communion wafer, baby puffs, cheese puffs, chiffon cake, and tofu pudding were generally rated at Level 5 Minced and Moist or above, remaining within the solid‐like levels of the IDDSI framework.

However, the descriptions of the final texture across all food samples were highly varied. Some foods, including milk chocolate and tofu pudding, received ratings spanning the entire range from Level 0 to Level 7 and included designations of “mixed consistencies.” “Mixed consistencies” were more prominent when rating cheese puffs. In most samples, one or two participants were undecided about the final texture and reported a consistency in between IDDSI levels, that is, between Levels 4 and 5 for tofu pudding and baby milk biscuit balls; between Levels 3 and 4 for milk chocolate and gelatin jelly; between Levels 2 and 3 for gelatin jelly; between Levels 5 and 6 for communion wafer; and between Levels 6 and 7 for cheese puff.

### 3.3 IDDSI Fork Pressure Test for Transitional Foods

Table 2 shows the results of the IDDSI fork pressure test conducted on the 10 food samples provided to the participants. Those that could be considered as passing the fork pressure test were milk biscuit balls, the two baked meringues, gelatin jelly, and tofu pudding. The communion wafer, baby puffs, cheese puffs, chiffon cake, and milk chocolate failed the fork pressure test.

**TABLE 2:** Appearance and observations of the IDDSI fork pressure test conducted on the food samples.

Sample	Dimensions	Initial	After 1 min with 1 mL of water	After fork pressure	IDDSI fork pressure test result	Comments
Baby milk biscuit ball	1.5 cm diameter × 1 cm height	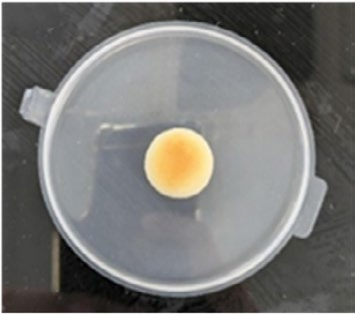	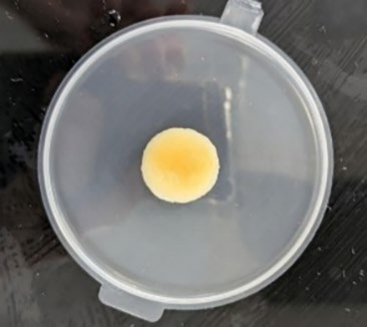	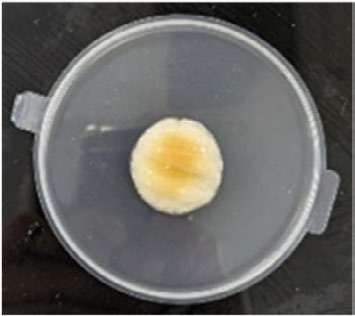	Pass	Absorbs water and deforms slightly when water is added (wrinkly appearance). Fork presses through easily and while it maintains its general shape, indentation of fork prongs can be seen.
Baked Meringue #1	1.5 cm diameter × 1 cm height	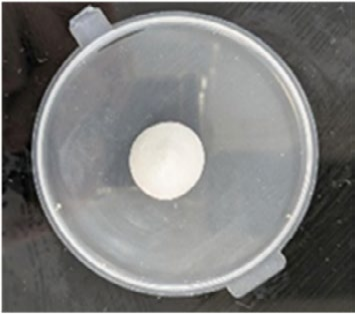	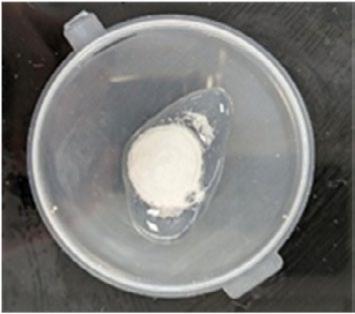	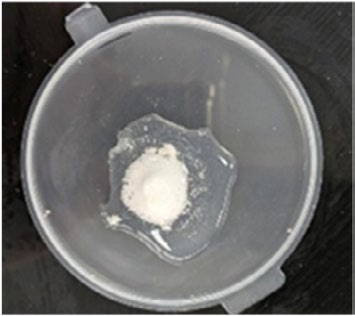	Pass	Partially dissolves when water is added. Little to no force required to press through the sample. Sticks to fork upon release of pressure. Final texture is a foamy paste.
Baked Meringue #2	1.5 cm diameter × 1 cm height	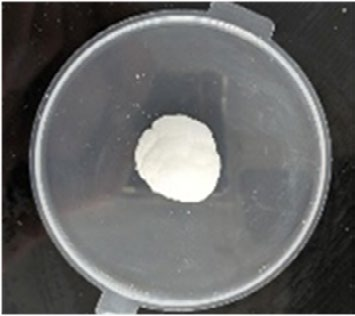	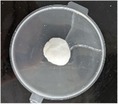	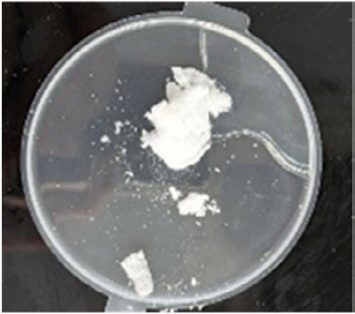	Pass	Water slides off. Still dry and brittle when pressing but force required is low enough to be able to be deformed. Sticks to fork upon release of pressure.
Communion wafer	Quadrant 1.5 cm radius × 0.2 cm height	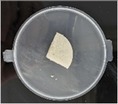	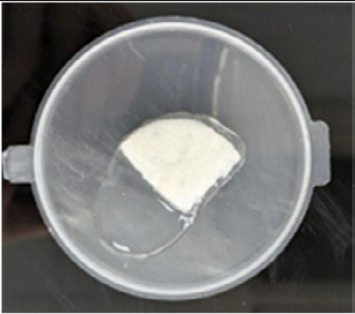	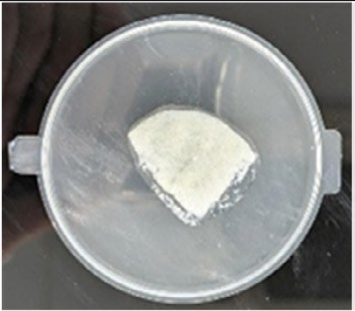	Fail	Absorbs water and swells slightly. Only slightly flattened after pressing. But no fracture and minimal indentation seen.
Baby puff	Pentagon with line segment 1.5 cm × 0.5 cm height	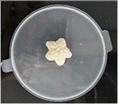	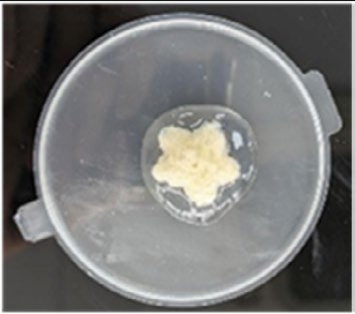	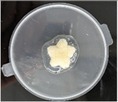	Fail	Absorbs water slightly. Flattened slightly upon pressing.
Cheese puff	1.5 cm × 1.5 cm × 0.5 cm height	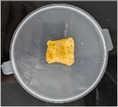	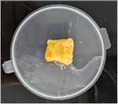	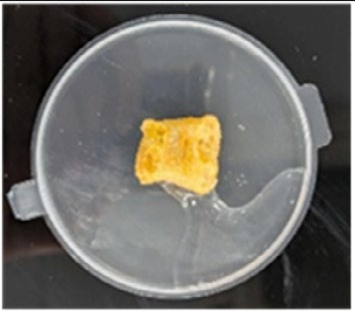	Fail	Water completely slides off. Does not deform upon pressing.
Chiffon cake	1.5 cm × 1.5 cm × 1.5 cm	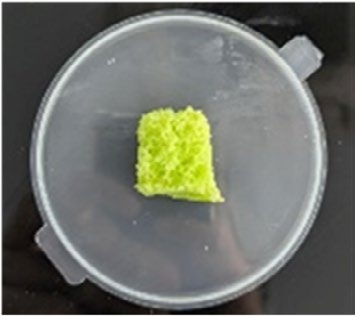	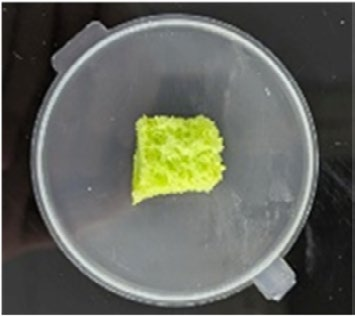	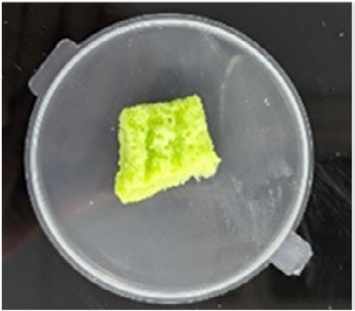	Fail	Absorbs water. Soft enough to be pressed halfway but not completely. Indentation of fork prongs can be seen upon the release of pressure but sample does not break apart.
Milk chocolate	1.5 cm × 1.5 cm × 0.9 cm height	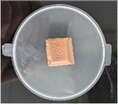	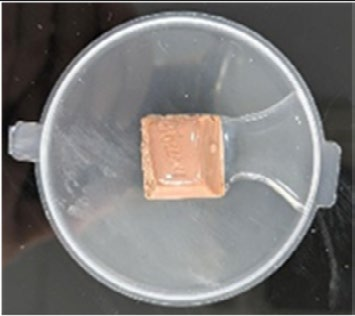	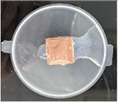	Fail	Does not absorb water. Does not deform upon pressing.
Gelatin jelly	1.5 cm diameter × 1 cm height	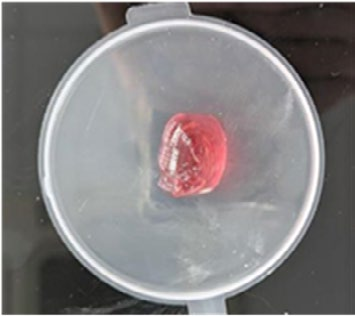	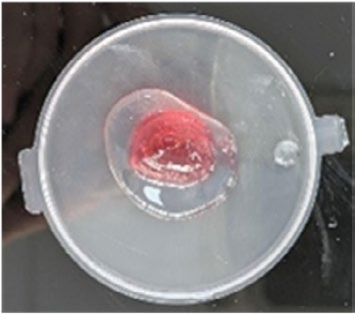	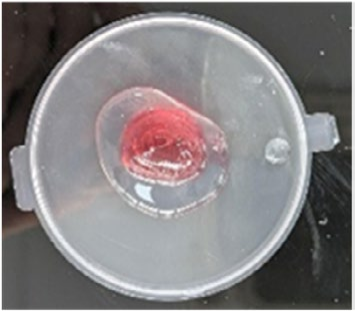	Pass	Does not absorb water. Little to no force required for fork to cut through the jelly into separate pieces. The separate pieces continue to stand in place unless moved apart by fork.
Tofu pudding	1.5 cm diameter × 1 cm height	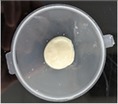	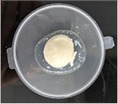	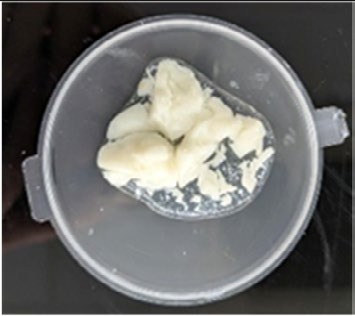	Pass	Does not absorb water. Little force required to deform and fracture. Some pieces stick to fork upon release of pressure.

The results reveal some divergence between the sample tasting in the focus group discussions and the outcomes of the fork pressure test. For instance, tofu pudding clearly passed the fork pressure test, yet most participants did not consider it as transitional. Conversely, milk chocolate failed the fork pressure test, although slightly more than half of the participants considered it transitional. Even for samples where tasting results and fork pressure tests aligned, differences emerged in the final texture ratings. For example, gelatin jelly was perceived by participants to have a final texture of Level 0 Thin Fluids (Figure 4), comparable to a thin liquid, but it retained a solid‐like form in the fork pressure test (Table 2).

### 3.4 Major Themes From Focus Group Discussion

#### 3.4.1 Definition and Understanding of Transitional Foods

##### 3.4.1.1 Limited Experience With Transitional Foods in Practice

When evaluating the list of transitional food examples compiled from an earlier online survey,^1^ participants highlighted potential confusion between transitional foods and transitional feeding, as they did not unanimously agree with all the cited examples. Among the participants, only one reported using transitional foods regularly as part of feeding therapy. This participant worked with the pediatric population in a private clinic. For the remaining participants, all of whom worked in hospital settings, the use of transitional foods was incidental rather than intentional.

I think I don't intentionally go like ’I'm going to use a transitional food. (Participant 101)

… I would say like similar, it's more like incidental rather than deliberate. (Participant 102)

##### 3.4.1.2 Change in Texture of Transitional Foods

In line with the definition outlined by the IDDSI framework, participants associated transitional foods with the idea of “change.” Throughout the discussion, commonly used terms to describe the change were “melt,” “dissolve,” “disappear,” “disintegrate,” “shatter,” “crumble,” “collapse,” “turn,” “transit,” “thin/watered down,” “breakup/broke down,” “transform” and “soluble.” Among these terms, “melt” was the most frequently used.

I think it's the ones that may be a bit more traditional … that fit the [IDDSI] definition bit more, will be obviously those that melt in the mouth right. So, the ice cream, ice chips, frozen yogurt. (Participant 102)

Participants determined that foods that melted or dissolved function as transitional foods, compared to those that transitioned purely by physical manipulation. Gelatin jelly and tofu pudding, both soft gels, illustrated the distinction. Participants generally rated the final texture of the gelatin jelly as Level 0 Thin fluids, while the tofu pudding was rated as Level 5 Minced and Moist diet.

Somehow this one just doesn't feel like a transitional food but I don't have the descriptors to describe exactly why. Other than the fact that the rest felt like they melted, …, whereas this one is just breaking down. (Participant 102)

Similarly, for the chiffon cake, participants noted that while there was a change in volume and texture, it was largely due to the mashing by the tongue as opposed to melting.

That's why I was keen to put no. Even though eventually it changed. I mean, if you mash anything hard enough with your tongue. (Participant 404)

##### 3.4.1.3 Vagueness of the Current Definition

Participants raised confusion with regard to the *degree* of change in texture required for it to be transitional. While the change for some foods was more significant, others were more minor, prompting participants to question the vagueness of the definition. As one participant stated,

… is the question like it changes state completely, that means from solid to liquid or solid to like nothing? Or can it still stay like solid but less solid? (Participant 402)

#### 3.4.2 Characteristics of Transitional Foods

##### 3.4.2.1 Textural Attributes

Several key textural attributes were identified by participants across focus group discussions.

- Transitional foods should not require significant force to break down. While the food could be hard and crispy initially, it may break down easily without much force required, such as the baby milk biscuit balls and the baked meringue samples.
- The food should remain relatively cohesive and should not spread out in the mouth, which would make it difficult to contain and control within the oral cavity. The ability to form a cohesive bolus that can be swallowed easily in a single swallow was highlighted as an ideal characteristic.
- The food should not be excessively sticky, as this would lead to adherence to the palate or other areas within the oral cavity, making it difficult to manipulate. Furthermore, a sticky bolus would impede swallowing and increase the likelihood of post‐swallow residue.
- The food should be smooth. A certain degree of graininess was acceptable, as in the case of baby milk biscuit balls, where the grains were small and soft. However, for foods such as the cheese puffs, participants encountered larger, hard grains that were abrasive and uncomfortable to swallow.
- For gel‐like products such as gelatin jelly and tofu pudding, these foods should not be excessively slippery, as this increases the risk of the food sliding back into the throat, potentially causing choking.

##### 3.4.2.2 Transitioning Evenly

Participants identified the requirement for foods that transitioned evenly, that is, where the entire food changes homogeneously from one texture to another. Based on the food samples tasted, few participants rated milk chocolate and cheese puffs as transitional foods (Figure 2). This was primarily due to the uneven transitions they exhibited. For milk chocolate, only the outer layer melted, while the inner portion remained solid. In the case of cheese puffs, participants noted that the outer “skin” was difficult to dissolve, and the final texture contained undissolvable “hard bits.” For both foods, multiple participants classified them as having “mixed consistencies” (Figure 5), due to the simultaneous presence of two textures, which posed safety risks. The presence of a mixed consistency was also the primary reason most participants did not consider tofu pudding a transitional food, despite it passing the IDDSI fork pressure test.

So somebody might have the whole spoonful in the mouth, then the drink doesn't meet the consistency. Yeah, the drink will then just start to flow to the back of the mouth when they are still trying to manipulate the [solid component of the tofu pudding] in the mouth, right? Then that's not too great for them. (Participant 301)

##### 3.4.2.3 Time Taken to Transition

A rapid transition time was consistently raised across discussions. Participants stated that they would more readily offer patients transitional foods that transitioned rapidly. Although longer‐transitioning foods, such as milk chocolate, might still be considered transitional, a key concern was a patient's ability to hold the food in the mouth for the necessary duration. When prompted for a specific time frame after evaluating all the samples, participants generally agreed on a period of 5–10 s. Based on the results in Figure 2, however, only the two baked meringue samples fell within this range.

##### 3.4.2.4 Size or Volume

Participants stressed the importance of smaller sizes or volumes. While this applied to the initial state of the food, the final volume of the bolus after it transitioned was also a key consideration.

“The volume is a lot more compared to the rest. In terms of volume for the others… a lot of them were like solid and then became liquid. Then all the air pockets' kind of gone, right. So I guess it appears a lot lesser in terms of the volume.” (Participant 401).

##### 3.4.2.5 Shape

Except for the participant who primarily worked with pediatric patients, the others did not specify shapes but favored finger foods. These foods could be picked up by patients to provide a tactile eating experience and encourage self‐feeding. The participant working with pediatric patients noted that stick‐like shapes would be useful for therapy purposes.

The issue with the Gerber puffs is [they are] so small that it's difficult for me to hold it at the side and hold it long enough for me to make sure that they use the jaw to chew on it… So nice ones are actually the prawn cracker kind … Yeah, thin and long. … that one is quite a nice shape because [it is] long enough for us at [sic] hold but not so long, until it's like too long. … So the child holds it, it's enough to go into the mouth. (Participant 303)

#### 3.4.3 Opportunities for Transitional Foods in Speech and Language Therapy Practice

##### 3.4.3.1 Opportunities in Patient Management

The focus group discussions, which had been grouped by institution or practice setting, revealed two key perspectives on the opportunities of transitional food in patient management.

###### 3.4.3.1.1 Individualisation and Product Stratification

The diversity in the transitional food characteristics and patients' swallowing abilities suggest a clear need for product stratification, enabling SLTs to match appropriate transitional foods to individual needs.

… hopefully there will be like two types of products … one for those who are really on tube feeding and all they can do is to manage their saliva swallows … Then another product range could be for those who are already eating but then need the challenge in order to upgrade their diet. (Participant 102)

###### 3.4.3.1.2 Institutionalized Implementation and Standardization

Despite the identified value in customisation, participants noted logistical challenges to implementing individualized approaches at scale. A solution suggested was developing a standard set of transitional foods with a narrower range of characteristics suitable for the general patient population.

But I guess if the goal of the transitional food is to allow as many patients to have it, then obviously something that ends around the Level 4—more patients will be suitable for it.” (Participant 404).

###### 3.4.3.1.3 Creating a Reference Scale for Transitional Foods

A standardized rating scale for transitional foods was proposed to help SLTs select suitable foods for patients.

For me, it'd be useful to have some rating scale to say the levels of how fast or how slow [the food transitions]. (Participant 303).

Other participants echoed this idea, suggesting tables or charts to characterize transition speed.

##### 3.4.3.2 Suggestions for Development

###### 3.4.3.2.1 Visually Appealing to Preserve Dignity

In addition to looking appetizing to consume, participants also emphasized the need for the food to look age appropriate. For example, participants highlighted that if the food were given to an elderly patient, it would ideally look like food meant for adults and not baby‐like food.

###### 3.4.3.2.2 Variety and Localized Flavors

Participants were enthusiastic about expanding the variety of flavors beyond what is typically available to patients. Common suggestions included incorporating local flavors or, more generally, stronger flavors that might appeal to elderly patients. For pediatric patients, the participant noted a lack of savory or umami options among the transitional foods currently used in therapy, suggesting that introducing such flavors could be beneficial for younger individuals.

###### 3.4.3.2.3 Meet Nutritional Concerns of Patients

While participants acknowledged that nutrition was not always a primary concern, they emphasized the importance of providing options that are nutritionally appropriate for patients. This is particularly relevant for elderly patients, many of whom suffer from multiple illnesses, including diabetes and sarcopenia. Consequently, developing transitional foods that are low in sugar and/or high in protein was among the suggestions provided by participants.

## 4 Discussion

The current definition of transitional foods is largely confined to the IDDSI framework documents (International Dysphagia Diet Standardisation Initiative 2019a, 2019b), though recent studies (Barewal et al. 2021; Larsen et al. 2024) have begun examining them more systematically. These investigations reveal both the potential benefits and challenges of transitional foods, particularly revealing the lack of clarity regarding their defining characteristics. This study extends those efforts by identifying key attributes that influence texture transition during oral processing.

A notable methodological feature of this study was the estimation of initial and preswallowing IDDSI levels by participants using a combination of visual and/or tactile observation (as samples were picked up or spooned) and in‐mouth sensory judgment, rather than formal IDDSI testing methods. This approach mirrors current clinical practice in Singapore, where SLTs typically do not apply IDDSI practical tests at bedside, but instead rely on expert observation and perception. While this method is not recommended by IDDSI and introduces subjectivity, it allowed us to capture real‐world clinical reasoning and attribute prioritization. Formal IDDSI tests were conducted separately by the research team and are reported independently to avoid biasing the qualitative discussion.

The variability observed across in‐mouth IDDSI level estimations is therefore expected, as foods continue to transition over time in the oral cavity and clinicians are not anchored to instrument outputs. The divergence between clinical estimations and IDDSI test outcomes is informative: it highlights gaps in current standards (e.g., the 1‐min observation period in IDDSI tests vs. typical clinical holding times) and opportunities to refine definitions for transitional foods.

The focus groups revealed that mechanical effort and time required for transition were critical attributes. Foods such as the baby milk biscuit balls and the baked meringue samples were initially firm but transitioned rapidly with minimal effort, whereas communion wafers and cheese puffs required greater mechanical effort and a longer oral processing time. Participants regarded rapid transition (5–10 s) as ideal, which is much shorter than the 1‐min duration used in the IDDSI fork pressure test. Comparisons between gelatin jelly and tofu pudding further demonstrated the association of a “melting” sensation with transitional foods. Gelatin melts near body temperature and was perceived as melting quickly to Level 0 (a thin liquid). In contrast, tofu pudding consists of a coagulated protein gel network that is insoluble at body temperatures and was not identified as a transitional food despite its texture changes, which resulted mainly from mechanical force breaking the gel into smaller pieces.

Another practical consideration was the initial portion size. Although a 1.5 cm × 1.5 cm bolus is consistent with IDDSI guidelines, which referenced mastication (Arai et al. 1992) and airway safety dimensions (Breatnach et al. 1984; Brodsky et al. 1996), participants noted that foods of this size could transition unevenly, as observed in the milk chocolate sample. Thus, the initial size should also reflect required textural changes and rate of transition. Ultimately, discussions about in‐mouth consistency were closely tied to the safety and acceptability of the transitional foods. Participants identified foods that remain cohesive (i.e., nonscattering), nonsticky yet nonslippery, and free from mixed consistencies throughout oral processing as most appropriate. Such requirements align with previous research emphasizing the importance of bolus cohesion and ease of oral clearance in dysphagia management (Bruno et al. 2025; Matsuo and Fujishima 2020; Nishinari et al. 2023). The results also suggest the need to better align the IDDSI fork pressure test with realistic oral processing conditions. Apart from a shorter waiting duration, the use of *warm* water (around body temperature) or warm amylase solution could offer a practical improvement in simulating the melting/dissolution behavior during oral processing arising from a range of mechanisms, from temperature‐induced phase change, dissolution of substance as well as salivary amylase breakdown.

Beyond physical properties, participants highlighted psychosocial and sensory factors. Transitional foods were seen as valuable for enhancing patient enjoyment and therapy engagement, consistent with findings from prior studies (Shune and Barewal 2022; Swan et al. 2015), yet current market options are mostly infant products. Participants advocated for adult‐oriented designs featuring savory and culturally familiar flavors, natural appearances, and recognizable forms. This aligns with evidence linking visual recognisability to improved acceptability (Lam et al. 2023).

Finally, participants viewed transitional foods as a flexible concept rather than a narrow category, with textures that can be tailored to dysphagia severity. This adaptability parallels recent developments in stabilized edible foams as reported by Hepper and Patterson (2023), suggesting the potential for transitional foods to become part of a broader and flexible approach to safe and enjoyable eating in dysphagia care.

## 5 Limitations and Future Directions

This research is limited to practicing SLTs in Singapore, which constrains the generalisability of findings across broader geographic and cultural contexts. The absence of patient perspectives means that the lived experience of individuals with dysphagia is not directly represented, and future research should include these voices to enrich understanding. As focus group discussions are qualitative by nature, it is difficult to quantify the prevalence of the extracted themes. Additionally, there was a predominance of participants from acute hospital settings, and expanding recruitment to include varied clinical and non‐clinical environments would strengthen future studies.

Methodologically, the fixed order of sample presentation may have introduced carryover effects. The assessment of later samples may have been influenced by palate adaptation, fatigue, or residue from preceding samples, slightly compromising the sensory independence of each data point. Future research employing quantitative sensory analysis should incorporate balanced randomization designs to control for this potential order bias. Furthermore, the use of subjective, in‐mouth IDDSI estimates limits the precision of texture classification. Reflexivity regarding researcher positionality and group dynamics was not systematically addressed, representing an area for methodological improvement.

Finally, while this study focuses on establishing foundational, objective safety attributes for transitional foods, it does not fully engage with the multisensory, phenomenological, and interdisciplinary dimensions of oral processing. Future research should incorporate perspectives from sensory studies, disability studies, medical anthropology, and neurology to develop a more holistic understanding of food–body interactions and the lived experience of dysphagia.

## 6 Conclusion

Transitional foods constitute a distinct category within the IDDSI framework due to their ability to change texture during oral processing with little to no mastication. However, defining the “suitable texture” of these foods is complex, as it depends on interdependent factors such as the food matrix, transition time, mode of transition, initial and final consistencies, and portion size.

Importantly, this work should be viewed as a first step toward a more critically sophisticated, interdisciplinary approach to understanding food, body, and sensory experience in dysphagia care. Insights from the current focus group discussions provide important clinical guidance on the key requirements that can facilitate the effective use of transitional foods in dysphagia management. Future research should build upon these clinical foundations by integrating phenomenological, sensory, and cultural perspectives, expanding participant diversity, and developing analytical frameworks that capture the complexity of embodied eating. Such efforts will be essential for optimizing both safety and quality of life for individuals with dysphagia.

## Author Contributions

Authors contributed to the conception and design of the study, data collection, analysis, and manuscript preparation. Specifically: **Seh Ling Kwong:** conceptualization, data curation, formal analysis, writing, review and editing. **Julia Mei Wan Lee:** conceptualization, data curation, formal analysis, writing. **Suk Meng Goh:** conceptualization, project administration, writing, review and editing. **Valerie Puay Cheng Lim:** conceptualization, formal analysis, review and editing. **Simeon Dobrev Stoyanov:** conceptualization, review and editing. **Katsuyoshi Nishinari:** review and editing. All authors have read and approved the final manuscript and agree with its submission to the Journal of Texture Studies. The manuscript has not been published elsewhere and is not under consideration by another journal.

## Funding

This work was supported by the Singapore Ministry of Education Academic Research fund Tier 2 Grant (MOE‐T2EP50223‐0017).

## Ethics Statement

This study was approved by the Institutional Review Board of Singapore Institute of Technology.

## Consent

Written informed consent was obtained from all study participants.

## Conflicts of Interest

The authors declare no conflicts of interest.

## Supporting information


**Data S1:** jtxs70064‐sup‐0001‐Appendix.docx.

## Data Availability

The data that support the findings of this study are available on request from the corresponding author. The data are not publicly available due to privacy or ethical restrictions.

## Endnotes

^1^
The online survey was performed to investigate the perspectives of speech and language therapists (SLTs) in Singapore regarding transitional foods and their use in dysphagia and feeding management. The results are reported in a separate manuscript.
