# Supplementary material for: Qualitative Insights From Practicing Speech and Language Therapists on Key Textural Attributes of Transitional Foods for Dysphagia Management
Source: J Texture Stud. 2026 Jan 30;57(1):e70064. doi: 10.1111/jtxs.70064 (PMC12857527; doi:10.1111/jtxs.70064)
Supplement: Supplementary file 1 — Data S1: jtxs70064‐sup‐0001‐Appendix.docx. [file JTXS-57-e70064-s001.docx]

# Appendix

Guiding questions used in focus group discussions:

| Category | Questions |
| --- | --- |
| Part 1: Introduction and understanding of transitional foods  4 questions | Review the definition of transitional foods (from IDDSI)  Review key aspects of the survey  According to IDDSI, a transitional food is described as “Food that starts as one texture (e.g. firm solid) and changes into another texture specifically when moisture (e.g. water or saliva) is applied, or when a change in temperature occurs (e.g. heating)”.  What are your thoughts on transitional foods?  Prompt: Do you have specific IDDSI level change in mind when you think of transitional foods?  Based on the survey, these are some examples of transitional foods cited from by the respondents (registered speech therapists in Singapore) from the survey, could you provide your thoughts on this list?  Prompts: Do you agree/disagree with any of the examples cited?  If transitional foods are used in practice, how would it be best presented?  Prompts: For example, in small pieces, etc. |
| Part 2: Sensory evaluation | Follow-up questions after tasting each sample  Would you consider this a transitional food? Why or why not?  Prompt: Please describe the characteristics of the food that make it a suitable/unsuitable transitional food.  Please describe how the food transitioned in the mouth.  Prompts:   - From what IDDSI level did it start and end with? - Did it melt? - Did you have to press it? - Did it transition after absorb moisture / dissolve?   After tasting all samples  What do you think is a suitable time frame for transition?  Do you have any other comments on the characteristics of transitional foods? |
| 3: Characteristics of transitional foods  5 questions | Based on the results of the survey, the top 3 factors of consideration for transitional foods were:   1. “swallowing safety”, 2. “amount of oral processing” for the texture 3. the “end consistency in mouth before swallowing”   Questions related to safety:  Could you elaborate on the meaning of swallowing safety?  How would you determine the swallowing safety of transitional foods?  What parameters would you propose be tested to ensure safety of transitional foods?  Prompts: For example, could you describe the:   - Parameters to test the initial texture? - Parameters to test the oral processing? - Parameters to test the final texture?   Questions related to oral processing:  What parameters would be important in the “amount of oral processing” that would be suitable for a transitional food?  Prompts: For example, could you describe the:   - Speed, force and amount of mastication (if any) - Speed, force and amount of tongue pressure (if any) - How long it stays in the mouth? - Texture of food e.g. stickiness, thickness, cohesiveness (How easily does the food stay together or break apart), graininess, or any others?   Questions related end consistency in mouth before swallowing:  Could you describe the “end texture in mouth before swallowing” that would be suitable for a transitional food?  Prompts: Could you specify the patient parameters? |
| 4: Recommendations | What kind of transitional foods would you like to see developed for your practice?  Prompts: For example, it could be:   - Types of food - Flavours - IDDSI level transition - Nutrition - Etc. |
